# The Model of an Ischemic Non-Healing Wound: Regeneration after Transplantation of a Living Skin

**DOI:** 10.17691/stm2023.15.5.01

**Published:** 2023-10-30

**Authors:** E.I. Morgun, K.K. Sukhinich, O.S. Rogovaya, E.A. Vorotelyak

**Affiliations:** PhD, Researcher, Laboratory of Cellular Biology; Koltzov Institute of Developmental Biology of Russian Academy of Sciences, 26 Vavilov St., Moscow, 119334, Russia; PhD, Researcher, Laboratory of Regeneration Problems; Koltzov Institute of Developmental Biology of Russian Academy of Sciences, 26 Vavilov St., Moscow, 119334, Russia; PhD, Senior Researcher, Laboratory of Cellular Biology; Koltzov Institute of Developmental Biology of Russian Academy of Sciences, 26 Vavilov St., Moscow, 119334, Russia; DSc, Correspondent Member of Russian Academy of Sciences, Head of the Laboratory of Cell Biology; Koltzov Institute of Developmental Biology of Russian Academy of Sciences, 26 Vavilov St., Moscow, 119334, Russia

**Keywords:** skin regeneration, ischemic non-healing wound, biomedical cell product, preclinical study of the skin equivalent, living skin equivalent

## Abstract

**The aim** was to evaluate the possibility of using our ischemic non-healing wound model for preclinical studies of biomedical cell products (BCP) during transplantation of a tissue-engineered construct.

**The tasks of the study** were to conduct the experiment on the transplantation of the tissue-engineered construct “living skin equivalent” (LSE) and select methods for determining the effectiveness of treating ischemic non-healing wounds during preclinical studies on the proposed model.

**Materials and Methods:**

The study was performed on 56 BALB/c mice divided into the following groups: “control” (n=19), “scaffold” (n=19), and “LSE” (n=18).

During the experiment, the histological, immunohistochemical methods and raster scanning optoacoustic mesoscopy (RSOM) technique were employed to compare the dynamics of regeneration of ischemic non-healing wound using LSE transplantation, collagen-hyaluronic film as a cell scaffold, and a non-treated wound.

**Results:**

Histology and immunohistochemistry have been found to be suitable to assess the effectiveness of treating ischemic non-healing wounds during preclinical investigations. The effect of LSE transplantation on infiltration of the wound bed with inflammatory cells, the formation of tissue in the wound bed zone, tissue condition at the wound margins, and angiogenesis has been studied. In addition, a new smoothing coefficient, i.e. the ratio of the thickness of the tissue-remodeling zone to the thickness of the dermis of the wound margins, has been proposed in the study. This coefficient makes it possible to assess the degree of filling the wound bed with the developing tissue. Its high value in the LSE group means that BCP transplantation influences the granulation tissue growth, prevents mechanical stress in the wound preventing thereby cosmetic defects.

Exploration of the regenerative processes has shown that the proposed model of the ischemic non-healing wound is suitable for preclinical studies of BCP.

## Introduction

Normally, regeneration of the cutaneous damage is completed by a full restoration of the skin structure and functions; however, when infection, hypoxia, or immune dysfunction are added, the wound may acquire the status of a chronic non-healing lesion [1]. These wounds are characterized by excessive inflammation, increased level of proteolytic activity, and delayed matrix deposition [2]. The regeneration stages of a non-healing wound are the same as for the normally healing wound, although with a significant delay in the inflammatory phase [3]. Diabetes, vascular insufficiency, exhaustion, elderly age, local infection, and compression-induced necrosis are referred to the factors of wound chronicity [3, 4]. Depending on the disease genesis, the following types of non-healing wound are distinguished: bed sores, diabetic ulcers, and ischemic non-healing wounds. The latter are resistant to conservative therapy.

One of the promising directions in managing nonhealing wounds is transplantation of biomedical cell products (BCP) of the skin equivalent type. This treatment contributes to effective remodeling of the granulation tissue and removal of a cosmetic defect, one of the wound chronicity sequalae [5]. The development of a model of ischemic non-healing wound adequate to human pathology is an integral part of researches directed towards the creation of BCP. Differences in the structure between the human skin and that of laboratory animals cause difficulties for the creation of similar models of ischemic non-healing wounds [6]. Researchers often use synthetic constructions and materials to make the process of regeneration in the animal model similar to the human pathology, reducing thereby the adequacy of the models. For example, presence of sutures in the immediate proximity to the wound causes a significant background during the work, therefore, the results are difficult to interpret. All this is one of the reasons of a great variety of models of ischemic non-healing wounds on the laboratory animals [7]. Presently, there are several patented models for the non-healing wounds in Russia, but there is no model, which would be perfectly suitable for conducting preclinical BCP studies.

**The aim** is to evaluate the possibility of using the ischemic non-healing wound model, developed in our laboratory, for preclinical studies of biomedical cell products during transplantation of a tissue-engineered construct.

**The tasks of the study are:** to conduct the experiment on transplantation of tissue-engineered construct “living skin equivalent” (LSE) representing an epidermal-mesenchymal layer on a carrier, and to select methods for determining the effectiveness of treating ischemic non-healing wounds during preclinical studies on the proposed model.

## Materials and Methods

### Cell cultures

The cell cultures of keratinocytes and mesenchymal stem cells (MSCs) obtained from Cell Culture Collection of Koltzov Institute of Developmental Biology of Russian Academy of Sciences (Moscow, Russia) were used for LSE preparation. Keratinocytes were cultivated on the DMEM/F-12 (PanEco, Russia) in the 1:1 ratio with fetal bovine serum (HyClone, USA), 5 pg/ml insulin (Sigma-Aldrich, USA), 10-6M isoproterenol (Sigma-Aldrich), 5 pg/ml transferrin (Sigma-Aldrich), 10 ng/ml EGF (Sigma-Aldrich), and 1% penicillin-streptomycin (Gibco, USA). MSCs were cultivated on the DMEM (PanEco) containing 10% of fetal bovine serum (HyClone) and 1% penicillin-streptomycin (Gibco).

To produce the biological skin equivalent (LSE), mouse keratinocytes and MSCs were cultivated on the collagen-hyaluronic film. Cells on the scaffold were ready to be transferred to the wound after 2-3 days.

### The work with animals

All manipulations with animals were done under general anesthesia in compliance with the Rules for the Work using Experimental Animals (Russia, 2010) and International Guiding Principles for Biomedical Research Involving Animals (CIOMS and ICLAS, 2012); the ethical principles of the European Convention for the Protection of Vertebrata used for Experimental and Other Specific Purposes (Strasburg, 2006) were strictly followed. The study was approved by the Bioethical Committee of Koltzov Institute of Developmental Biology of Russian Academy of Sciences (protocols No.23 of November 15, 2018 and No.28 of September 5, 2019). Animals were housed with free access to food and water. The study was performed on 56 BALB/c mice.

The mice were divided into the following groups: “control” (n=19), “scaffold” (n=19), and “LSE” (n=18). Before manipulations, the animals underwent general anesthesia with Avertin. Fur was removed in the area of the operation field with a hair removal cream (Veet, Canada). After depilation, a 30*10-mm rectangle was marked on the mouse skin, in the center of which a full-thickness circular opening with 5-7 mm in diameter was excised. Next, full-thickness parallel skin incisions were done along the marked lines and all large vessels were cut off the flap. Bleeding was arrested by applying hydrogen peroxide to the ligated vessels. The flap margins were sutured, the wound was washed, and Tegaderm™ plaster (Germany) was applied.

Transplantation of the scaffold and LSE in the appropriate groups was performed by applying these materials on the wound bed of the animals. After the operation, the wound was covered with a Tegaderm™ plaster.

On days 3-5, the wounds of mice in the control, scaffold, and LSE groups were washed with a sterile 0.1% solution of gentamycin on DPBS (PanEco). The animals were withdrawn from the experiment on days 5, 7, 14, and 21 by narcosis overdosage.

### Biomaterial preparation

The biomaterial for histological investigations was fixed in 10% formaldehyde (Biovitrum, Sweden). The biomaterial for immunohistochemical investigations was placed into the OCT Cryomount gel (HistoLab, Sweden) and frozen in liquid nitrogen.

### Histological investigation

The material embedded into paraffin blocks was used for histological investigations. Histological sections were obtained using the Microm HM 430 microtome (Thermo Fisher Scientific, USA). Preparations were stained with hematoxylin-eosin and Mallory’s trichrome stain.

### Immunohistochemical staining

The cryosections were prepared using the standard technique on the CM1900 cryostat (Leica Microsystems, Germany). Fixation was done with 4% paraformaldehyde. The block-solution, in which antibodies were diluted, contained 5% serum of the animal producing secondary antibodies. Preparations were incubated with primary antibodies overnight. Further, the secondary antibodies and DAPI solution were applied on the preparations, which were placed into the BrightMount/Plus medium (Abcam, UK). The following antibodies were used in the work: rabbit primary monoclonal antibodies against Krt14 (1:200; Ab197893; Abcam) and rat primary monoclonal antibodies against CD31 (1:100; Ab56299; Abcam), and also goat anti-rat secondary antibodies AlexaFluor 488 (1:600; Ab150157; Abcam) and donkey anti-rat secondary antibodies AlexaFluor 488 (1:500; A-21206; Invitrogen, USA).

Preparations were viewed and photographed using microscopes BZ-9000E (Keyence, Japan) and IX73 (Olympus, Japan).

### Raster scanning optoacoustic mesoscopy

The dynamics of the wound healing process was studied using the RSOM Explorer P50 mesoscope (iThera Medical, Germany).

### Morphometry and statistical analysis

The morphometric analysis was performed by means of ImageJ program. Data were analyzed in the Excel program using R programming language and RStudio environment. The Kruskal-Wallis nonparametric test for multiple comparisons was applied for comparative data analysis. Comparisons between the groups were performed by means of Dunn’s test. Differences were considered statistically significant at p<0.05.

## Results and Discussion

During preliminary experiments, the model of a nonhealing wound, which reproduced ischemic conditions and was similar to human pathology, has been developed in our laboratory [8]. However, it had some shortages, which hindered technically investigations connected with preclinical testing of BCP. For example, during flap formation, excessive bleeding to the wound bed was observed; besides, on days 7-14 the wounds suppurated. Moreover, the developed model did not have a covering, which would protect BCP in the wound bed. Therefore, some procedures were performed to optimize the model: bleeding from the ligated vessels was arrested with hydrogen oxide, the wound was washed with a sterile 1% gentamycin solution on DPBS during the operation and postoperative care, and the Tegaderm™ plaster was applied to the wound (see the Table).

**Table:** Problems in preclinical studies of biomedical cell products arising during modeling an ischemic non-healing wound and ways to solve them in the proposed model

Problems	Ways of solving
Active bleeding in the wound	Treatment of ligated vessels with hydrogen peroxide with subsequent washing out of this solution by a 0.1% gentamycin solution on DPBS
Presence of auxiliary constructions (sutures, rings) at the wound margins, which allows wound healing modeling by scarring and epithelialization rather than by contraction	Rings and sutures at the wound margins are absent; contraction is prohibited by the sutures located at the edges of the ischemic flap at a distance from the wound
Wound suppuration; usage of cytotoxic antiseptic solutions	Application of 0.1% gentamycin solution on DPBS prevents wound suppuration and does not cause cell death in the biomedical cell products
Wound localization complicating the performance of the experiment due to the animal self-injury or difficult access for the experimenter	Creating the wound in the interscapular area which is difficult to access by the animal but convenient for the experimental work
Absence of the wound covering or usage of undurable and non-transparent dressing makes dynamic observation of the wound condition difficult	Application of the Tegaderm™ plaster (which is transparent and convenient in use) allows observation of the wound condition

In order to assess the suitability of the proposed model, an experiment for BCP testing was carried out. It included the transplantation of the tissue-engineered construct of LSE or the transplantation of the scaffold kept in the conditions similar to LSE. No transplantation was done to the mice of the control group. The Tegaderm™ plaster was used to cover the wounds of mice in all groups.

The histological, immunohistochemical, and raster scanning optoacoustic mesoscopy (RSOM) methods were chosen to evaluate the effectiveness of treating ischemic non-healing wounds during preclinical studies on the proposed model and to compare the wound conditions.

### Characteristic of the wound process by histological and immunohistochemical methods

The histological analysis has shown that the inflammatory phase in the above model lasts up to 5 days from getting the injury. At this stage, the wound bed of all mice was characterized by infiltration with inflammatory cells. On day 7-14 of wound healing, a proliferative phase was observed. During this phase, gradual formation of the granulation tissue was noted in the wound bed. On day 21, there came the phase of re-epithelialization and remodeling. The majority of animal wounds in all groups were characterized by mature scars and formed epithelium. The histological analysis allowed us to characterize in detail and compare the condition of all animal wounds according to the stages of wound healing. The following parameters were proposed as the criteria for assessing the effectivity of wound treatment using BCP: vascularization of the wound bed, infiltration of the wound bed with inflammatory cells, the state of the tissue-remodeling zone in the wound bed, as well as the number of hair follicles (HF) at the wound edges [9].

### Inflammation phase

**Figure 1.**
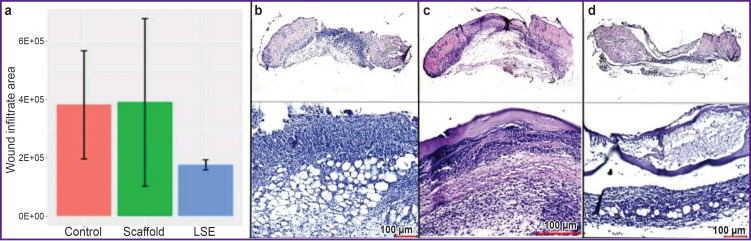
Regeneration of the non-healing wound at the stage of inflammation: (a) area of inflammatory infiltrate in the control, scaffold, and LSE groups; wound condition of the mouse on day 5, staining with hematoxylin-eosin: (b) "control”, (c) "scaffold”, (d) "LSE”

As it has been mentioned above, the lag in the wound process at the phase of inflammation and excessive inflammatory response underlie the acquisition of the non-healing status by the wound [10]. Excessive infiltration of the wound bed with neutrophils is a key factor in the development of chronic inflammation and may be considered as a histological biomarker of chronic non-healing wounds [4]. On day 5, the wound bed of many mice from the control group was filled with inflammatory infiltrate. Migration of the inflammatory cells to the adipose tissue and fascia under the wound bed and at the wound margins was noted. Besides, significant degradation of epidermis and derma at the wound margins and areas of the dead cells have been found (Figure 1 (b)). The wounds of many mice from the scaffold group had a similar histological picture (Figure 1 (c)). At the same time, moderate infiltration of the wound bed, adipose tissue, and fascia of the entire wound area with inflammatory cells was observed in the majority of mice from the LSE group (Figure 1 (d)). The comparison of the examined groups has demonstrated the tendency to the reduction in the area of the inflammatory infiltrate of the wound bed in the mice of the LSE group (Figure 1 (a)). Hence, we may suggest an immunomodulating effect of LSE owing to the MSCs in its composition.

### Proliferation phase

#### Granulation tissue

In the period of proliferation, maturation of the granulation tissue occurred in the mice of all groups. However, a number of essential differences in the formation of the granulation tissue was noted between the mice from the control and LSE groups.

It is known that non-healing wounds are characterized by impairment in granulation tissue formation due to various reasons. For example, the overproduction of reactive oxygen species by neutrophils causes damage to extracellular matrix [4]. TNF-a, inducing collagenase activity, is also supposed to inhibit normal scarring [11-13].

**Figure 2.**
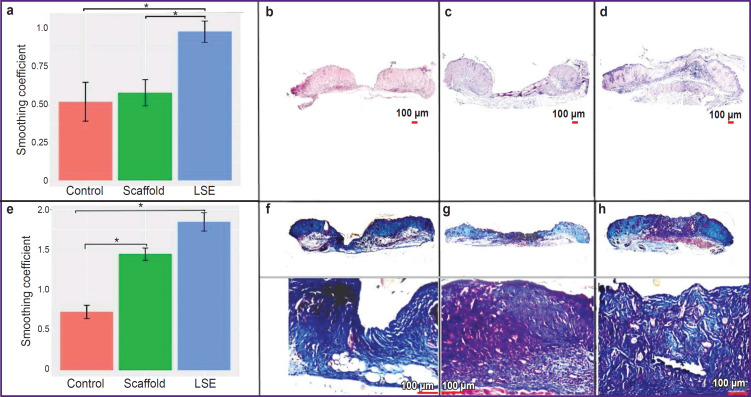
Regeneration of the non-healing wound at the stage of proliferation: smoothing coefficient in the control, scaffold, and LSE groups on day 7 (a) and day 14 (e), * p<0.05; wound condition of the mouse on day 7, staining with hematoxylin-eosin: (b) “control”, (c) “scaffold”, (d) “LSE”; wound condition of the mouse on day 14, Mallory’s staining: (f) “control”, (g) “scaffold”, (h) “LSE”

In the control group, imitating a non-healing wound without treatment, a great difference was noted on the histological preparations between the thickness of dermis at the margins and the thickness of the tissueremodeling zone at the wound center in the majority of animals. The wound margins rose significantly above the wound bed on day 7 of the experiment (Figure 2 (b)). In the scaffold group, a similar picture was observed (Figure 2 (c)). On day 7, the thickness of the tissueremodeling zone in the wound bed in the majority of animals of the LSE group almost reached the wound margins (Figure 2 (d)).

On day 14, the thickness of the tissue-remodeling zone in many animals of the control group was still significantly less than that of the dermis at the wound margins (Figure 2 (f)); in the scaffold group, the thickness of the tissue-remodeling zone partially or fully reached the wound margins (Figure 2 (g)); in the LSE group, the tissue-remodeling zone filled completely the wound bed by width and height in the majority of animals (Figure 2 (h)). The morphological middle of the wound in the mice of the control group on day 14 was characterized by a scanty amount of the tissue, while in the groups “scaffold” and “LSE” it was plentiful; the granulation tissue in the LSE group had well visualized fibers. To describe the effectiveness of treatment with LSE transplantation in respect of the effect on granulation, the term “smoothing coefficient” was introduced, which determines the ratio of the thickness of the tissue-remodeling zone to the thickness of the dermis of the wound margins. In the LSE group, the smoothing coefficient was statistically significantly higher than that in the groups “scaffold” and “control” on day 7, which spoke of the efficacy of LSE transplantation for removing the defect of the granulation tissue, which may result in cosmetic problems (Figure 2 (a)). On day 14, the smoothing coefficients in the groups “LSE” and “scaffold” were statistically significantly higher than in the control group, and at the same time did not differ statistically significantly from each other (Figure 2 (e)).

#### Tissue condition at the wound margins. Hair follicles: death and regeneration

On day 7, degenerative changes in the wound margin tissue continued in many animals of all groups. Due to the individual differences between the rates of regeneration, diverse variants of margin conditions were observed at a given point in time: moderate degradation interfollicular epidermis, dermis, and HF at the wound margins; mass death of these structures with subsequent formation of a scab; partial tissue regeneration. HF regeneration is known to occur in various ongoing processes: regeneration in microinjury, regeneration in case of the partial HF loss, wound-induced hair neogenesis typical for large full-thickness wounds in rodents [14], and also wound- induced anagen [15]. In our work, it was not possible to identify the type of regeneration during which the HF restoration occurred in the process of ischemic chronic wound healing due to technical difficulties. In this connection, we use the term “HF regeneration” not categorizing its type.

**Figure 3.**
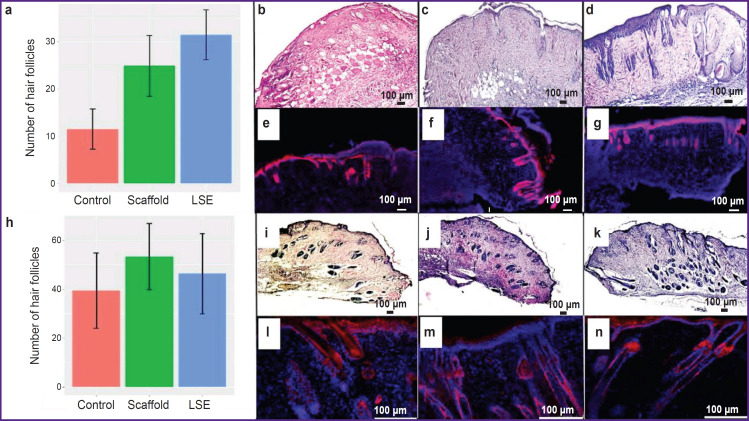
Regeneration of the non-healing wound at the stage of proliferation: average number of hair follicles per wound margin in the control, scaffold, and LSE groups on day 7 (a) and 14 (h); condition of the mouse wound margin on day 7, staining with hematoxylin-eosin: (b) “control”, (c) “scaffold”, (d) “LSE”; staining with antibodies to Krt14, nuclei are additionally stained with DAPI: (e) “control”, (f) “scaffold”, (g) “LSE”; condition of the mouse wound margin on day 14, staining with hematoxylin-eosin: (i) “control”, (j) “scaffold”, (k) “LSE”; staining with antibodies to Krt14, nuclei are additionally stained with DAPI: (l) “control”, (m) “scaffold”, (n) “LSE”

In our experiment, certain differences in the dynamics of degenerative processes in HF, their death and regeneration were noted in the animals of control and LSE groups. Thus, in many mice from the control group, there was noticed a mass HF death at the wound margins. The HF cells were characterized by marked degenerative changes and kariolysis (Figure 3 (b)). At the same time, in the LSE group, many HF located at the wound margins had either normal morphology or moderate signs of degeneration (Figure 3 (d)). An average amount of HF per a wound margin increased in the LSE group relative to the control group. It may speak of the fact that LSE contributed to the preservation of HF or accelerates the process of their regeneration (Figure 3 (a)).

On day 14, HF of the majority of wounds had normal morphology; the number of HF in the groups did not differ statistically significantly. Thus, the rate of HF regeneration in all three groups became equal (Figure 3 (h)).

#### Intensity of angiogenesis

**Figure 4.**
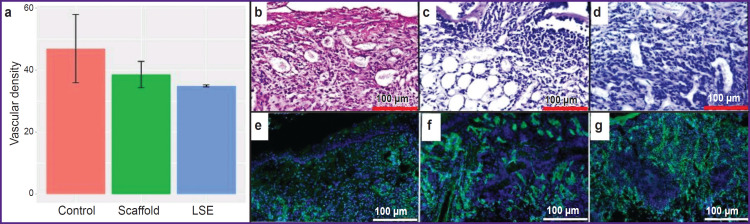
Regeneration of the non-healing wound at the stage of proliferation: (a) vascular density in the mice wound bed in the control, scaffold, and LSE groups; condition of the mouse wound bed on day 7, staining with hematoxylin-eosin: (b) “control”, (c) “scaffold”, (d) “LSE”; staining with antibodies to CD31, nuclei are additionally stained with DAPI: (e) “control”, (f) “scaffold”, (g) “LSE”

The quantitative analysis has revealed absence of statistically significant differences between the density of vessels in the wound bed in the mice of the three groups on day 7. Consequently, LSE does not influence angiogenesis (Figure 4).

#### Scar remodeling and reepithelialization

**Figure 5.**
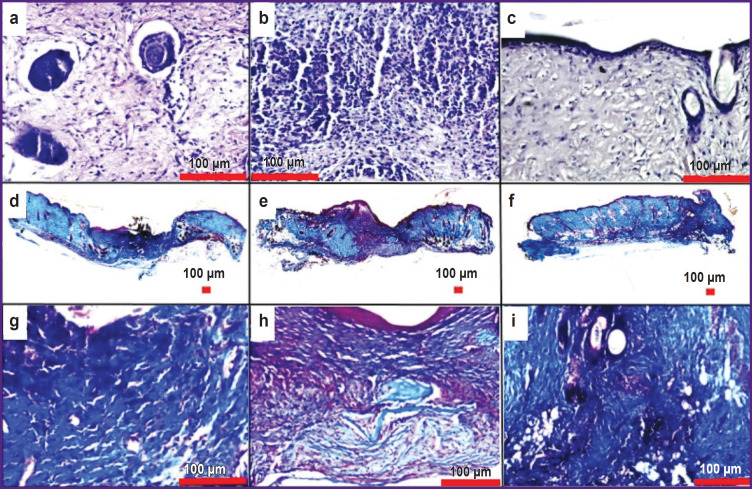
Regeneration of the non-healing wound at the stage of scar remodeling and reepithelialization: condition of the mouse wound bed in the examined groups on day 21, staining with hematoxylin- eosin: (a) “control”, mature scar with HF; (b) “scaffold”, infiltration with inflammatory cells, predominance of the cell component over fibers; (c) “LSE”, mature scar with HF; Mallory’s staining: (d, g) “control”; (e, h) “scaffold”; (f, i) “LSE”

On day 21, the majority of the wounds in the animals of all groups were characterized by scar formation and reepithelialization (Figure 5). In many mice, fibers prevailed over the cell component in the scar tissue; besides, HF in the phase of a mature anagen were also observed. Therefore, the conclusion may be drawn on the full completion of the wound regeneration process. However, in some mice, the cell component prevailed over fibers in the wound bed, infiltration with inflammatory cells was noted, meaning that the process of wound healing is not finished. Both variants of wound healing were present in the mice of all groups. Thus, the rate of regeneration in the groups on day 21 became equal.

### Characteristics of the wound process using raster scanning optoacoustic mesoscopy

Raster scanning optoacoustic mesoscopy is the latest non-invasive technology, which allows one to get high-resolution images. RSOM is scanning the skin area of interest with a transducer in parallel with illumination with a bundle of optic fibers. Optoacoustic waves generated in the tissue in response to the pulsed illumination are fixed; the acquired image is a 3D propagation of the absorbed light in the tissue. The reconstructed images demonstrate the distribution of melanin and hemoglobin in the epidermis and dermis making it possible to obtain images of microvascular skin system at the depth of 1-2 mm [16].

**Figure 6.**
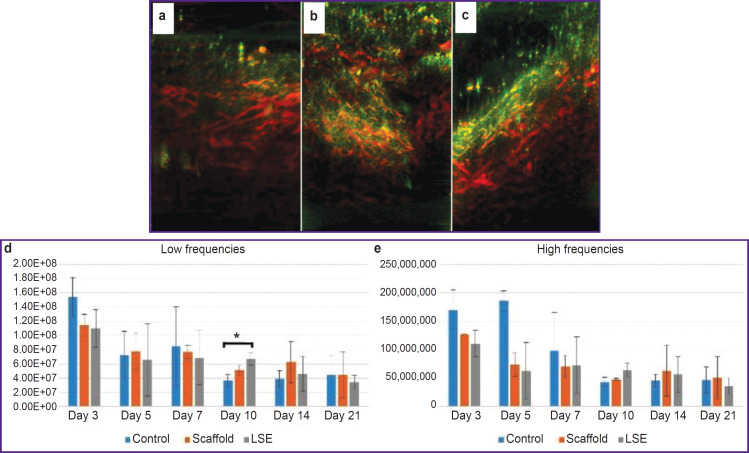
Investigation of the wound bed using raster scanning optoacoustic mesoscopy: 3-dimensional reconstruction of the mouse vessels on day 21 in the control (a), scaffold (b), and LSE (c) groups; low-frequency signal is shown by a red color, high-frequency signal is green; signal intensity at low (d) and high (e) frequencies, * p<0.05

Since RSOM is successfully used in the investigations of skin pathologies such as psoriasis and atopic eczema [17], this method was used in the present study to evaluate the dynamics of wound blood flow as a criterion of successful BCP transplantation. Measurements were performed on days 3, 5, 7, 10, 14, and 21. As a result of the experiment, it has been established that on day 10 the intensity of the signal at low frequencies in the LSE group exceeded statistically significantly that in the control group (Figure 6 (d)). This fact may be interpreted in favor of the idea that LSE contributes to angiogenesis during wound healing.

In the process of examining the wound bed by means of RSOM, a number of technical difficulties have been experienced. Thus, the material, from which the scaffold for LSE cells was constructed, hindered the experiment and created a significant background. Besides, the transplant interfered with the observation of the underlying microvasculature condition, whereas the wound of mice without transplantation remained open, therefore, the comparison of the results obtained appeared to be incorrect. Not only the transplant but the plaster as well, removal of which created the risk of wound infection, prevented measurements. Besides, during measurements, the animal experiencing the load caused by blood loss had to remain under general anesthesia for a long time, which negatively influenced its state and often resulted in death. All this suggests the conclusion that RSOM cannot be recommended for the evaluation of the effectiveness of BCP transplantation during preclinical studies.

## Conclusion

Exploration of the regenerative processes has shown that the proposed model of the ischemic non-healing wound is suitable for preclinical studies of biomedical cell products. The evaluation of the parameters such as wound infiltration with inflammatory cells, the condition of the tissue-remodeling zone, a number of hair follicles, vascularization of the wound bed according to the appropriate stages of wound healing using histological and immunohistochemical methods is quite adequate and suitable for preclinical studies of biomedical cell products on the examined model. Additionally, a new indicator, a smoothing coefficient, expressed as a ratio of the thickness of the tissueremodeling zone to the thickness of the wound margin dermis, allowed to evaluate the degree of occupation of the wound bed with the developing tissue. Its high value in the LSE group means that transplantation of the biomedical cell products influences the properties of fibroblasts, hampers mechanical strain in the wound, and, consequently, prevents formation of a cosmetic defect. This indicator will make it possible to assess the condition of the tissue-remodeling zone in the wound bed with the transplantation and without it, and to predict thereby the effectiveness of biomedical cell products to eliminate cosmetic defects.

The evaluation of the wound blood flow by raster scanning optoacoustic mesoscopy cannot be recommended for preclinical studies of biomedical cell products on the proposed model due to its specific features.
